# Does social need fulfillment moderate the association between socioeconomic status and health risk behaviours during pregnancy?

**DOI:** 10.1093/eurpub/ckae102

**Published:** 2024-06-18

**Authors:** Stella Weiland, Danielle E M C Jansen, Henk Groen, Dorien R de Jong, Jan Jaap H M Erwich, Marjolein Y Berger, Annemieke Hoek, Lilian L Peters

**Affiliations:** Department of Primary and Long-term Care, University of Groningen, University Medical Center Groningen, Groningen, The Netherlands; Midwifery Academy Amsterdam Groningen, InHolland, Groningen, The Netherlands; Amsterdam UMC Location Vrije Universiteit Amsterdam, Midwifery Science, Amsterdam, The Netherlands; Department of Primary and Long-term Care, University of Groningen, University Medical Center Groningen, Groningen, The Netherlands; Department of Sociology and Interuniversity Center for Social Science Theory and Methodology (ICS), University of Groningen, Groningen, The Netherlands; Department of Epidemiology, University of Groningen, University Medical Center Groningen, Groningen, The Netherlands; Department of Primary and Long-term Care, University of Groningen, University Medical Center Groningen, Groningen, The Netherlands; Midwifery Academy Amsterdam Groningen, InHolland, Groningen, The Netherlands; Amsterdam UMC Location Vrije Universiteit Amsterdam, Midwifery Science, Amsterdam, The Netherlands; Department of Obstetrics and Gynecology, University of Groningen, University Medical Center Groningen, Groningen, The Netherlands; Department of Primary and Long-term Care, University of Groningen, University Medical Center Groningen, Groningen, The Netherlands; Department of Obstetrics and Gynecology, University of Groningen, University Medical Center Groningen, Groningen, The Netherlands; Department of Primary and Long-term Care, University of Groningen, University Medical Center Groningen, Groningen, The Netherlands; Midwifery Academy Amsterdam Groningen, InHolland, Groningen, The Netherlands; Amsterdam UMC Location Vrije Universiteit Amsterdam, Midwifery Science, Amsterdam, The Netherlands

## Abstract

Socioeconomic differences in health risk behaviours during pregnancy may be influenced by social relations. In this study, we aimed to investigate if social need fulfillment moderates the association between socioeconomic status (SES) and health risk behaviours (smoking and/or alcohol consumption) during pregnancy. We used baseline data from the Lifelines Cohort Study merged with data from the Lifelines Reproductive Origin of Adult Health and Disease (ROAHD) cohort. Education level was used to determine SES, categorized into low, middle, and high, with middle SES as the reference category. Social need fulfillment was taken as indicator for social relations and was measured with the validated Social Production Function Instrument for the Level of Well-being scale. The dependent variable was smoking and/or alcohol consumption during pregnancy. Univariable and multivariable logistic regression analysis was conducted to assess the association of SES and social need fulfillment with health risk behaviours and to test for effect modification. We included 1107 pregnant women. The results showed that women with a high SES had statistically significantly lower odds of health risk behaviours during pregnancy. The interaction effect between SES and social need fulfillment on health risk behaviours was not statistically significant, indicating that no moderation effect is present. The results indicate that social need fulfillment does not modify the effect of SES on health risk behaviours during pregnancy. However, in literature, social relations are identified as an important influence on health risk behaviours. More research is needed to identify which measure of social relations is the most relevant regarding the association with health risk behaviours.

## Introduction

In the Netherlands, 8% of women smoke during (part of) their pregnancy and 2.6% consume alcohol during pregnancy [1]. Women who smoke or consume alcohol tend to engage in other health risk behaviours during pregnancy, such as an unhealthy diet and inadequate folic acid intake [2]. These health risk behaviours are associated with adverse outcomes, such as low birthweight, preterm birth, and miscarriage [3, 4].

Health risk behaviours during pregnancy are associated with socioeconomic status (SES), where women with a lower SES are at greater risk of continuation of unhealthy behaviours during pregnancy compared to women with higher SES [5]. Socioeconomic differences in health behaviours during pregnancy may be explained by psychosocial stress and available resources [2, 6]. Women with a lower SES tend to have fewer resources, such as income, knowledge, and social support, compared to women with higher SES [7]. Lacking these resources increases psychosocial stress and may make individuals at greater risk to turn to unhealthy behaviours to cope with psychosocial stress [7]. One study reported that stress is a mediator between lower SES and postpartum smoking relapse [8]. Studies on the pathways between SES and the consumption of alcohol during pregnancy are scarce; one study reports that women consume alcohol during pregnancy as a mechanism to cope with stress [9].

Social relations may positively help manage psychosocial stress. Previous studies stated that social relations play an important role in socioeconomic differences in health behaviours [7, 10]. Social relations may function as a buffer against psychosocial stress and thereby have a positive influence on health behaviours [11, 12]. On the other hand, social relations might negatively influence health behaviours. Women who smoke often have many smokers in their social networks, which influences women’s attitudes towards smoking during pregnancy [13].

The mechanism could be different for pregnant women compared to the general population, because pregnant women are also responsible for the health of their unborn child. Putatively, this new responsibility for the unborn makes women more dependent on social support to cope with general and pregnancy-related stress [8]. There are limited studies that examined the moderating effect of social relations on the association between SES and health behaviours during pregnancy. One previous study investigated pathways between SES and smoking during pregnancy [14]. This study did not report evidence in favour of the moderating effect of social relations. This study used the quality of the primary intimate relationship as operationalization for social relationships and had smoking in the third trimester as outcome [14]. The results of another study, not performed among pregnant women, indicated that social support may act as a buffer for stress and problem drinking [15].

**Figure 1.**
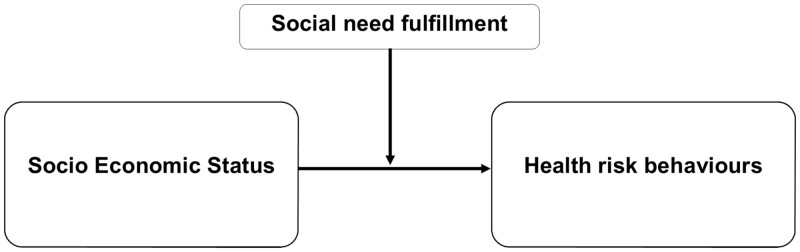
Hypothesized moderation effect of social need fulfillment on socioeconomic status and health risk behaviours.

Another way of operationalization of social relationships is social need fulfillment as defined within the Social Production Function (SPF) theory [16, 17]. According to this theory, all humans are motivated to optimize their social needs (e.g. the need for social support and friendship), and therefore achieve psychosocial well-being [16, 17]. Social need fulfillment relates to the social aspect of the SPF theory. According to the SPF theory, individuals have three basic social needs and goals: affection, behavioural confirmation, and status. Affection refers to the need to be loved, liked, and accepted. Behavioural confirmation refers to the need of feeling that important others think that you are a good person or that you are doing the ‘right’ thing. Status refers to the need of feeling that you have an influence, are taken seriously, or are known for your skills and achievements. The fulfillment of these three social needs leads to social well-being [16, 17]. We will use the SPF theory, as measured with the validated Social Production Function Instrument for the Level of Well-being (SPF-IL) questionnaire, to investigate the following research question: does social need fulfillment moderate the association between SES and health risk behaviours (smoking and/or alcohol consumption) during pregnancy? By answering this question, we aim to understand the role of social need fulfillment in the association between SES and health behaviours during pregnancy (Fig. 1).

## Methods

### Design and study population

In this study, we used baseline data from the original Lifelines Cohort Study merged with data from the Lifelines Reproductive Origin of Adult Health and Disease (ROAHD) cohort, which is nested in the original Lifelines Cohort Study. Lifelines is a large representative population-based cohort study and a biobank in the northern provinces of the Netherlands with the aim to investigate risk factors for multifactorial diseases [18, 19]. Recruitment for the Lifelines Cohort Study was performed between 2006 and 2013 (*N* = 167 729). Inhabitants of the northern part of the Netherlands were invited to participate through their general practitioner. The Lifelines Cohort Study collects different types of data: biomaterial (e.g. urine, blood), physical examination (e.g. pulmonary function, blood pressure), and questionnaires. All participants gave informed consent before they underwent a physical examination. Participants completed multiple questionnaires about general characteristics (i.e. education, work), health (i.e. healthcare use, health status), lifestyle and environment (i.e. smoking, nutrition), and psychosocial parameters (i.e. social relations assessed with the SPF-IL questionnaire, stress) [18].

The aim of the Lifelines-ROAHD cohort is to investigate the health of women in their reproductive age (20–45 years). Data were collected in the period September 2017 until Mach 2018. From the original Lifelines Cohort, 30 712 women of reproductive age were eligible to participate in the Lifelines-ROAHD Cohort. In total, 5933 (19.3%) women completed the online questionnaire for the Lifelines-ROAHD study. After excluding women who did not complete essential parts of the questionnaire, in total, 5412 women participated in the Lifelines-ROAHD study, of which 2604 women had not been pregnant and 2808 women had experienced 6158 pregnancies and 5068 births [20]. The Lifelines-ROAHD questionnaire comprised items on women’s health: menstrual cycle characteristics, menopause, contraceptive use, fertility problems, and assisted reproduction treatments. If women had experienced a pregnancy, they also received items about conception, medication use during pregnancy, lifestyle (smoking during pregnancy, second-hand smoking, alcohol use, weight gain), course of pregnancy, and pregnancy outcomes. If women had given birth, they received additional questions about the onset of delivery, mode of birth, birth outcomes, health outcomes for mothers, and infant health problems [20].

The current study focuses on the population of women in the northern parts of the Netherlands (age ≥ 18 years). We included the women who had experienced at least one pregnancy independent of pregnancy outcome. From the Lifelines-ROAHD cohort, we selected women with data about health behaviours during pregnancy around two years prior to and after the Lifelines baseline assessment, including the social need fulfillment questionnaire (SPF-IL). If women were pregnant more than once in this time interval, we selected the pregnancy that occurred closest to the baseline assessment. Women were excluded from the analyses when they had missing data on education level or more than two missing values on the SPF-IL questionnaire.

## Measurements

### Demographics

Demographic characteristics that were collected included maternal age, marital status (single or in a relationship), migration background (Dutch or non-Dutch), and SES operationalized as the respondents highest attained education level. We chose education level as measure for SES because education is related to non-material resources such as knowledge and health literacy, which facilitate a healthy lifestyle [21]. In addition, there is an association between education level with smoking and alcohol consumption [1]. We categorized education level into a SES score according to the guideline of Statistics Netherlands, which is based on the International Standardized Classification of Education (ISCEI) [22]. We categorized a low SES as being lower educated (having finished primary education or lower- or preparatory secondary education), a middle SES as being middle educated (having finished middle or higher secondary education), and a high SES as being higher educated (having finished higher vocational education or university). Furthermore, we collected data about lifestyle characteristics, such as body mass index (BMI, kg/m^2^, overweight and obesity), and pregnancy characteristics, such as planned pregnancy (yes or no) and parity (nulliparous or multiparous).

### Health risk behaviours during pregnancy

Health behaviours included in this study were smoking behaviour and/or alcohol consumption during pregnancy. In the ROAHD questionnaire, women were asked whether they had smoked or consumed alcohol during the pregnancy. They could either answer: (1) ‘yes, during part of the pregnancy’, (2) ‘yes, during the entire pregnancy’, or (2) ‘no’. The two variables (smoking and alcohol consumption) were combined into one variable. If women had either smoked or consumed alcohol during a part or their entire pregnancy, they were categorized as ‘yes’. If women never smoked or consumed alcohol during pregnancy, they were categorized as ‘no’.

### Social need fulfillment

Social need fulfillment was assessed using the validated nine-item SPF-IL scale [17, 23]. The SPF-IL scale contains questions relating to the three social needs during the past three months: affection (three items), behavioural confirmation (three items), and status (three items). For example, respondents were asked: ‘do you feel that people really love you?’ (affection), ‘do others appreciate the things you do?’ (behavioural confirmation), and ‘do people find you an influential person?’ (status). The items were scored on a 4-point Likert scale ranging from 0 (never) to 3 (always,) resulting in a summed scale score with a maximum of 27. A higher score indicates higher social need fulfillment.

### Other control variables

We pre-identified possible confounders. We identified maternal age (continuous) as possible confounder from the demographic characteristics [24]. From pregnancy characteristics, we included partners in consecutive pregnancies (dichotomized as same partner compared to multiple partners), parity [24], and planned pregnancy (dichotomous) [25] as possible confounders. With respect to parameters of lifestyle, we included BMI (continuous) [24], second-hand smoke exposure (dichotomous), physical illness (dichotomous), and psychological illness (dichotomous) as confounders. We decided to take second-hand smoke exposure into account because it is associated with smoking during pregnancy [26]. Because the presence of physical or psychological disease may influence health behaviours [27, 28], we also controlled for the presence of physical or psychological diseases prior to pregnancy, as measured in the Lifelines baseline assessment. Having a chronic physical disease may positively influence health behaviours (i.e. not smoking or drinking), while psychological diseases may negatively influence health behaviours [27, 28]. The most common physical diseases with a high burden among women in the reproductive age are hypertension and migraine [29, 30]. If women had one or both chronic diseases, they were categorized as having at least one physical disease (dichotomous). Common psychological diseases are depression, social phobia, agoraphobia, panic disorder, (other) anxiety disorder, and manic-depressive disorder [31]. If women had one or more of these psychological diseases, they were categorized as having at least one psychological disease (dichotomous).

### Statistical analysis

To report baseline characteristics, we used descriptive statistics. To assess differences between subgroups who differed on SES, we used Chi-Square tests and Fisher-Freeman-Halton exact test, where appropriate. Differences between the SES-groups on the SPF-IL score were tested with Kruskal–Wallis test, since the data were not normally distributed.

Univariable and multivariable logistic regression was used to assess the hypothesized effect modification of social need fulfillment (as measured with the total score of the SPF-IL) on the association between SES and health risk behaviours during pregnancy. First, we performed a univariable logistic analysis with SES as independent variable and health risk behaviours as dependent variable. Middle SES was taken as the reference category. We also performed a univariable logistic analysis for the SPF-IL score on health risk behaviours. Second, we performed multivariable logistic regression analyses where we assessed the association between SES and the total SPF-IL score on health risk behaviours (model 1). Subsequently, we added the confounders and the interaction terms of SES and the total SPF-IL score (model 2). For the selection of confounders, we performed a multivariable analysis for each potential confounder in the full model on health risk behaviours separately. If the relative change of associations of the primary exposure was greater than 10% after adding the possible confounder to the model, we concluded that there was confounding [32]. To be able to interpret the interaction effects, we reported the betas with the *P* values and calculated the odds ratios (ORs) for the interaction terms by putting the median SPF-IL score and the median plus one standard deviation score in the model. For each model, we calculated ORs with corresponding 95% confidence intervals (95% CI). All data were analysed in SPSS version 26.0 (SPSS Inc., Chicago, IL, USA). A *P* values ≤ 0.05 was considered statistically significant.

## Results

### Participants

From the 2808 women of the Lifelines-ROAHD cohort who experienced at least one pregnancy, in total, 1701 women were excluded because: they did not experience a pregnancy two years before or after completing the baseline questionnaire (*n* = 1694), had more than two missing values on the SPF-IL (*n* = 3), or because they had missing values on education level (*n* = 4). Our final study population consisted of 1107 women with a mean age (SD) of 31.7 (3.8) years. A minority of 5.0% had a low SES, 36.3% a middle SES, and 58.7% a high SES (Table 1). The SES-subgroups showed statistically significant differences on maternal age, number of partners in consecutive pregnancies, parity, BMI, smoking behaviour, second-hand smoke exposure, and presence of physical disease and psychological disease. Compared to women with a middle and high SES, women with a low SES were more often multiparous, obese, and smoked more often during pregnancy. Women with a low SES had a higher prevalence of health risk behaviours (smoking and/or alcohol consumption) during pregnancy compared with women with a middle or high SES. Women with a low SES had statistically significantly lower scores on the SPF-IL (*P* ≤ 0.001), compared with women with a middle or high SES; median scores (interquartile range) were 24.0 (21.0–25.0), 25.0 (23.0–27.0), and 25.0 (24.0–27.0) respectively, indicating they had a lower social need fulfillment.

**Table 1.** Population characteristics of women in the Lifelines Cohort Study and Lifelines-ROAHD by socioeconomic status

	Total population	Low SES	Middle SES	High SES	Statistical differences between SES groups
	*N* = 1107	*n* = 55 (5.0%)	*n* = 402 (36.3%)	*n* = 650 (58.7%)	
	*N* (%)^a^	*n* (%)	*n* (%)	*n* (%)	*P* value
Maternal characteristics					
Maternal age (in years) during pregnancy					0.05
18–30	333 (30.1)	24 (43.6)	136 (33.8)	173 (26.6)	
31–35	518 (46.8)	19 (34.5)	184 (45.8)	315 (48.5)	
≥36	256 (23.1)	12 (21.8)	82 (20.4)	162 (24.9)	
Migration background					0.26
Dutch	1058 (95.6)	55 (100)	382 (95.0)	621 (95.5)	
Non-Dutch	49 (4.4)	–	20 (5.0)	29 (4.5)	
Marital status					0.60
Single	37 (3.3)	≤10 (≤0.2)	≤10 (≤0.02)	20 (3.1)	
In a relationship	741 (66.9)	38 (69.1)	274 (68.2)	429 (66.0)	
Missing	329 (29.7)	14 (25.5)	114 (28.4)	201 (30.9)	
Pregnancy characteristics					
Partners consecutive pregnancies					0.03
Same partner	881 (79.6)	45 (81.8)	308 (76.6)	528 (81.2)	
Two or more partners	70 (6.3)	≤10 (≤0.2)	35 (8.7)	31 (4.8)	
Missing	156 (14.1)	≤10 (≤0.2)	59 (14.7)	91 (14.0)	
Parity					≤0.001
Nulliparous	497 (44.9)	14 (25.5)	167 (41.5)	316 (48.6)	
Multiparous	610 (55.1)	41 (74.5)	235 (58.5)	334 (51.4)	
Planned pregnancy					0.08
Yes	960 (86.7)	43 (78.2)	344 (85.6)	573 (88.2)	
No	147 (13.3)	12 (21.8)	58 (14.4)	77 (11.8)	
Lifestyle characteristics					
BMI^b^					≤0.001
Overweight	285 (25.7)	17 (30.9)	123 (30.6)	145 (22.3)	
Obesity	136 (12.3)	13 (23.6)	67 (16.7)	56 (8.6)	
Smoking behaviour					≤0.001
No	1026 (92.7)	43 (78.2)	357 (88.8)	626 (96.3)	
Yes	81 (7.3)	12 (21.8)	45 (11.2)	24 (3.7)	
Second-hand smoke exposure^c^					≤0.001
Yes	50 (4.5)	≤10 (≤0.2)	31 (7.7)	15 (2.3)	
Alcohol consumption					0.59
Yes	66 (6.0)	≤10 (≤0.2)	20 (5.0)	43 (6.6)	
Health risk behaviours^d^					≤0.001
No	984 (88.9)	43 (78.2)	346 (86.1)	595 (91.5)	
Yes	123 (11.1)	12 (21.8)	56 (13.9)	55 (8.5)	
Presence of physical disease					
No	787 (71.1)	39 (70.9)	265 (65.9)	483 (74.3)	0.01
Yes	320 (28.9)	16 (29.1)	137 (34.1)	167 (25.7)	
Presence of psychological disease					
No	947 (85.5)	43 (78.2)	328 (81.6)	576 (88.6)	0.01
Yes	160 (14.5)	12 (21.8)	74 (18.4)	74 (11.4)	

a Percentages may not add up to 100% due to rounding.

b BMI classified as: overweight (25.0–29.9), obesity (>30).

c As response to the question: Are there other household members smoking inside the house during pregnancy?

d Composite outcome of smoking behaviour and/or alcohol consumption during pregnancy. The numbers of smoking behaviour and alcohol consumption do not add up to the composite outcome since some participants both smoke and consume alcohol.

### Moderation effect

The univariable logistic analysis showed that women with a high SES had statistically significantly lower odds of health risk behaviours during pregnancy (OR 0.57, 95% CI 0.39–0.85) (Table 2). Social need fulfillment was not statistically significantly associated with health risk behaviours (OR 0.96, 95% CI 0.91–1.02).

**Table 2.** Univariable and multivariable logistic regression models on health risks behaviours during pregnancy

	Univariable logistic regression	Multivariable logistic regression	
	Health risk behaviour	Health risk behaviour	Health risk behaviour
	OR (95% CI)	Model 1 OR (95% CI)	Model 2 aOR (95% CI)^a^
Socioeconomic status			
Middle	Ref.	Ref.	Ref.
Low	1.72 (0.86–3.47)	1.67 (0.83–3.38)	0.01 (0.00–9.32)
High	**0.57 (0.39–0.85)** ^b^	**0.58 (0.39–0.86)**	0.35 (0.01–11.70)
SPF-IL score^c^	0.96 (0.91–1.02)	0.98 (0.92–1.04)	0.95 (0.86–1.05)

a Adjusted for the dichotomous variables second-hand smoke exposure, partners in consecutive pregnancies and planned pregnancy.

b Numbers in bold indicate statistically significant results (*P* ≤ 0.05).

c The total SPF-IL score.

In the multivariable logistic regression analysis, including SES and the total social need fulfillment score (model 1), there was a significant association between high SES and health risk behaviours during pregnancy (OR 0.58, 95% CI 0.39–0.86). After adding the confounders and interaction terms (model 2), there were no statistically significant associations between SES, the SPF-IL score, and health risks behaviours. The interaction effect between SES and social need fulfillment on health risk behaviours was not statistically significant, indicating that no moderation effect is present (Table 2). The betas for the interaction effects for low SES and the SPF-IL score and high SES and the SPF-IL score were 0.207 (p = 0.33) and 0.029 (p = 0.66), respectively. For the low SES group, the odds for health risk behaviours during pregnancy were 1.66 times higher if the SPF-IL score increased with one standard deviation, in comparison with the middle SES group. For the high SES group, the odds for health risk behaviours during pregnancy were 0.93 times lower if the SPF-IL score increased with one standard deviation, in comparison with the middle SES group.

## Discussion

### Main findings

In this study, we investigated if social need fulfillment moderates the relationship between SES and health risk behaviours (smoking and/or consuming alcohol) during pregnancy. In our study population, women with a low, middle, and high SES showed statistically significant differences on maternal age, number of partners in consecutive pregnancies, parity, BMI, smoking behaviour, second-hand smoke exposure, health risk behaviours, presence of physical disease, and presence of psychological disease. Women with a low SES had a statistically significant lower social need fulfillment score than women with a middle or high SES. Women with a high SES had lower odds of smoking and/or consuming alcohol during pregnancy compared with women with a middle SES. However, this association did not remain after adjustment for confounders. The interaction effects between SES and social need fulfillment on health risk behaviours were not statistically significant, indicating that no moderation effect is present.

### Strengths and limitations

A strength of this study is the richness of the available data about women’s pregnancies, such as BMI of women, data about second-hand smoke exposure, and partners in consecutive pregnancies. The prevalence of smoking in this cohort is comparable to the prevalence in the Dutch population, and the observed prevalence of alcohol consumption is higher than reported in a previous study [1]. However, smoking and alcohol consumption might still be underreported, since these were self-reported measures. Furthermore, the data were retrospectively collected, which might have caused a recall bias. Another limitation is the relatively high proportion of women with a high SES in the data compared to the general Dutch population [33]. In our study, 59% of the women had a high SES, whereas in the Dutch population, this is 40% [33]. The overrepresentation of women with a high SES and the relative low number of women with a low SES influences the generalizability of the results. Lastly, we combined smoking and/or alcohol consumption because of power issues. The relative low number of women with a low SES (*n* = 55) could explain why we did not find significant results for this group.

### Interpretation

Our results show that women with a low SES have a lower social need fulfillment score, indicating that they feel less affection, behavioural confirmation, and status compared with women with a middle or high SES [17, 23]. This is in accordance with a previous study which reports that women with a low SES have fewer resources, which influences their health behaviours [7]. Although women with a low SES tend to have a less healthy lifestyle [5], we did not find a statistically significant association between a low SES and health risk behaviours during pregnancy. However, the high SES group did have statistically significant lower odds of health risk behaviours during pregnancy compared with the middle SES group. It could be that the association between SES and health behaviours differs across groups [34]. Other factors than SES influence health behaviours during pregnancy and could be a reason for differences in associations between SES groups [34]. For example, years of smoking, confidence, self-efficacy, and health concerns are associated with health risk behaviours [35]. Furthermore, the statistically significant association between high SES and health risk behaviours disappeared after adjusting for second-hand smoke exposure, partners in consecutive pregnancies, and planned pregnancy. This means that these variables likely explain part of the association between SES and health risk behaviours.

Contrary to the results of previous studies, we did not find a statistically significant association between social need fulfillment and health risks behaviours during pregnancy [12, 36]. This could be related to the influence of pregnancy on women’s lifestyle. Women are more likely to change their lifestyle during pregnancy because they are responsible for the health of the unborn child [37]. Despite the importance of social relations [8], pregnancy itself could be a main factor for women to change their health behaviours [37]. Another potential reason could be the differences in measures for social relations that were used. Previous studies used the quality of the intimate relationship or the perceived availability of interpersonal resources as measures for social relations [11, 14]. Especially support from a partner seems to be an important factor, which is associated with smoking cessation and a reduced likelihood of binge drinking during pregnancy [26, 38].

The measure used for social relations might also explain why we did not find a moderation effect, indicating that social need fulfillment does not influence the association between SES and health risk behaviours during pregnancy. We hypothesized that social need fulfillment could act as a buffer for stress and therefor positively influence health risk behaviours [10–12]. However, the type of social support that functions as a buffer for stress might depend on the source and type of stress [12]. In literature, there is no conclusive evidence about how social relations influence health behaviours [12, 14]. While some studies argue for a moderation effect, another study found evidence for a mediating effect with stress and social relations as mediators [11, 12, 14]. Future studies should particularly focus on the measure of social relations that influences health behaviours during pregnancy. Insight into the influence of social relations on health behaviours during pregnancy has important implications for interventions. Interventions aiming to improve social support are effective by improving social well-being and might be beneficial for health risk behaviours of pregnant women [39]. Furthermore, interventions that focused on peer support seem to be promising for addressing substance use during pregnancy [40].

## Conclusion

In this study, we aimed to investigate if social need fulfillment moderates the relationship between SES and health risk behaviours during pregnancy. The results indicate that social need fulfillment does not modify the effect of SES on health risk behaviours during pregnancy. However, in existing literature, social relations are identified as an important influence on health risk behaviour. More research is needed to understand the pathways and to identify which measure of social relations is the most relevant regarding the association with health risk behaviours.

## Data Availability

The data that support the findings of this study are available from the Lifelines Cohort Study, but restrictions apply to the availability of these data, which were used under license for the current study, and so are not publicly available. Data are, however, available from the authors upon reasonable request and with permission of the Lifelines Cohort Study.
